# Sexual violence, mental health, and suicidality—Results from a survey in cooperation with idea‐driven organizations and their social media platform followers

**DOI:** 10.1002/hsr2.973

**Published:** 2022-12-02

**Authors:** Axel C. Carlsson, Ulrika Owen, Gita Rajan

**Affiliations:** ^1^ Division of Family Medicine and Primary Care, Department of Neurobiology, Care Sciences and Society (NVS) Karolinska Institutet Huddinge Sweden; ^2^ Academic Primary Health Care Centre, Stockholm Region Stockholm Sweden; ^3^ Public Health Agency of Sweden Solna Sweden

**Keywords:** adverse childhood experiences, childhood trauma, mental health, self‐harm

## Abstract

**Aims:**

We specifically aimed to study if individuals who have been subjected to sexual violence more frequently report psychiatric diagnoses and suicide attempts. We also aimed to investigate if individuals who had been sexually violated reported more or less childhood experiences of family environment with alcohol problems, suicide attempts, and domestic violence than those without sexual violations.

**Methods:**

In 2019, the nongovernment organization World of no sexual abuse (WONSA) collected data through a web‐based survey. The survey was shared via websites of idea‐driven organizations working with victims of sexual violence, on social media and through email lists. A total of 4831 individuals participated and 49% answered all questions.

**Results:**

Of the participants exposed to penetrating sexual violence, 49% stated that they had or had been diagnosed with depression, compared with 16% in the group not exposed to sexual violence. Similar findings were found for anxiety: 45% versus 12%; fatigue syndrome 28% versus 9%; post traumatic stress disorder 30% versus <0.1% and suicide attempts, 29% versus 3%. More participants in the group exposed to sexual violence had grown up in families with alcohol problems, suicide attempts, or where they have witnessed violence.

**Conclusion:**

Steps should be taken to adapt the national suicide prevention strategy to the association between sexual violence and suicide attempts, which has been so clearly demonstrated earlier in both international and national studies, and which is again shown in the present study.

## 1 INTRODUCTION

Adverse childhood experiences (ACEs)^1^ is a term that defines 10 potentially traumatic interpersonal events in childhood. The ACE events that have been shown to be most strongly linked to both the development of illness and to premature death in adulthood are growing up in families with substance violence, mental illness, suicide, guardians in prison, witnessing violence between guardians, being neglected as a child, being abandoned through separation, exposure to sexual, physical, or psychological violence.

The prevalence of both ACEs and sexual violence in adulthood are susceptible risk factors for mental illness, suicide attempts, and addiction problems. Sexual violence is also one of the potentially traumatic events that seem to increase the risk of further traumatic events. The risk of new events in survivors of sexual violence is called revictimization and is a phenomenon associated with further ill health.^2^, ^3^, ^4^, ^5^, ^6^, ^7^, ^8^, ^9^, ^10^ The associations between ACEs, sexual violence, suicide attempts, and mental health are strong, yet complex. More knowledge about how these factors affect each other is important for public health to unravel.

Deficiencies in the existing routines in healthcare to register and document the prevalence of ACEs and sexual violence in patient records make it difficult to extract reliable data on the associations between exposure to ACEs, and sexual violence with suicidality and poor mental health.^7^ Furthermore, Sweden has received criticism from the EU for the health care system's shortcomings in terms of documentation and follow‐up of sexual violence in healthcare.^11^ A logical step to increase the knowledge about the aforementioned associations would be to include questions about ACEs and sexual violence in national health surveys.

Our hypothesis was that answers from a questionnaire sent from an organization with knowledge of sexual violence could provide increased knowledge about the association between sexual violence, abuse, and suicide attempts. With knowledge about these associations, it is possible to develop and target prevention activities. The overarching aim was to investigate whether and how data from a survey disseminated through idea‐driven organizations' social media platforms can increase knowledge about how the occurrence of sexual violence, with or without other childhood traumas, may affect the occurrence of diagnoses related to mental health, and suicide attempts. We specifically aimed to study if individuals who have been subjected to sexual violence more frequently report psychiatric diagnoses and suicide attempts. We also aimed to investigate if individuals who had been sexually violated reported growing up in family environments with alcohol‐ and drug problems, suicidality, or domestic violence more frequently than those not sexually violated.

## 2 METHODS

Between March 13, 2019, and May 17, 2019, the nongovernment organization World of no sexual abuse (WONSA) collected data through a web‐based survey. The survey was developed by WONSA and was made available online by the company Alstra, using a software tool for online questionnaires called Survey Generator. The survey was shared as a digital link by idea‐driven organizations on social media platforms such as Instagram, Facebook, and LinkedIn. The survey was also available on the websites of these organizations, and through their email lists. It was optional to participate as well as to answer the various questions. The participants could proceed in the questionnaire without having answered all the questions and could cancel the participation at any time. Only adults were allowed to participate, and both people with and without experience of sexual violence were welcome to participate. The online questionnaire was open for 73 days. All data was submitted by Alstra to WONSA in an excel document. A total of 4831 individuals logged in to the survey, and 49% answered all questions (*n* = 2367).

The main study sample in the present study included all participants with answers on all the questions about gender (female, male, nonbinary), exposure to sexual violence (yes, no, do not know) and suicide attempts (yes, no), *n* = 2958. Participants with answers to the questions on suicide attempts and exposure to penetrating sexual violence were analyzed and compared with the entire group exposed to sexual violence. To make use of as much data as possible on the subgroup exposed to penetrating sexual violence, it was analyzed separately regardless of whether they had responded to the gender question, *n* = 1500.

### 2.1 The online survey

The survey started with questions about gender, age and level of education, as well as about exposure to any type of sexual violence and suicide attempts. For gender, three possible answers were stated; “woman”, “man,” or “other.” The category “other” is referred to hereafter as “nonbinary.”

The question of exposure to sexual violence was formulated as: “Have you ever been subjected to sexual abuse or any form of sexual harassment?” with the answer options “Yes,” “No,” or “I do not know.” The question about suicidality was asked as: “Have you ever tried to take your life?” with the answer options “Yes,” “No but I have had thoughts,” or “No.”

The participants who reported exposure to sexual violence received additional questions to determine the type of sexual violence that had been used with the answer options “without physical contact,” “with physical contact,” or “with penetration of body opening.”

The participants also answered questions about the occurrence of a number of diagnoses and symptoms of diagnoses, as well as about assumptions about themselves. The present study focuses on diagnoses concerning mental illness, namely: depression, anxiety, post traumatic stress disorder (PTSD), and fatigue syndrome. The chosen diagnoses were based partly on previous data on connections to ACEs and sexual violence, and partly on experiences from clinicians at the WONSA's specialist clinic.^7^, ^8^, ^9^, ^10^

Finally, exposure to different ACEs was also part of the questionnaire.

### 2.2 Statistical analysis

Descriptive statistics are shown as numbers and proportions. The results are presented in tables for the whole group and stratified by gender. Data on the characteristic of the study group are presented as a percentage, in total and broken down by gender.

## 3 RESULTS

To avoid backward identification, the characteristics of the 27 individuals who identified themselves as nonbinary were omitted from the tables. The characteristics of all other participants are shown in Table 1. Only 7%, of the included participants identified themselves as men leaving 93% of the participants identifying themselves as women. Most of the respondents were in the age group 26–45 years (60%), with about 20% in the group 18–25 and 46–65 years, respectively. The highest level of education was high school for 22%, postsecondary education for 17% and university education for 59% of the respondents. Among the participants, 88% were exposed to sexual violence. Gender‐stratified statistics showed that 100% of the nonbinary, 91% of the women, and 40% of the men reported exposure to sexual violence, and for 65% of the participants, the sexual violence had started in childhood.

**Table 1:** Baseline characteristics

	Women *n* = 2754 (93%)	Men, *n* = 177 (6%)	Total, *n =* 2958 (100%)
**Education level**	**Women**, *n* = **2683 (93%)**	**Men**, *n* = **173 (6%)**	**Total**, *n* = **2883 (100%)**
Elementary school	62 (2%)	7 (4%)	69 (2%)
High school	593 (22%)	33 (19%)	634 (22%)
Professional education after high school	436 (16%)	39 (22%)	479 (17%)
University	1592 (60%)	94 (54%)	1701 (60%)

Age groups	Women, *n* = 2740 (93%)	Men, *n* = 171 (6%)	Total, *n* *=* 2938 (100%)
18–25	553 (20%)	18 (10%)	577 (20%)
26–45	1641 (60%)	92 (54%)	1753 (60%)
46–65	519 (19%)	60 (35%)	580 (20%)

Exposure to sexual violence	Women, *n* = 2754 (93%)	Men, *n* = 177 (6%)	Total, *n =* 2958 (100%)
Yes	2507 (91%)	72 (40%)	2606 (88%)
No	192 (7%)	99 (56%)	291 (10%)
Do not know	55 (2%)	6 (3%)	61 (2%)
Child sexual abuse (CSA)	1626 (65%)	36 (50%)	1682 (64%)

Reported psychiatric disorders and suicide attempts are shown in Table 2. The biggest differences were found between those exposed to penetrating sexual violence and to those without exposure to sexual violence, with those exposed to nonpenetrating sexual violence being in the middle indicating a dose effect. As many as 49% of those who had been subjected to penetrating sexual violence stated that they had or had been diagnosed with depression, compared with 16% in the group not exposed. Anxiety was reported by 45% of participants exposed to penetrating sexual violence compared with 12% in the group not exposed, and fatigue syndrome was reported among 28% of participants exposed to penetrating sexual violence versus only 9% in the group not exposed. PTSD was reported by 30% and 29% reported that they had carried out at least one suicide attempt compared with <0.1% having PTSD, and with 3% having committed at least one suicide attempt among not exposed.

**Table 2:** Reported diagnoses of psychiatric disorders and suicide attempts by exposure to sexual violence

	All types of sexual violence, *n* = 2606 (100%)	Exposed to penetrating sexual violence, *n* = 1500 (100%)	Not exposed to sexual violence, *n* = 291 (*n* = 100%)
Depression	1018 (39%)	741 (49%)	47 (16%)
Anxiety	904 (35%)	682 (45%)	36 (12%)
Fatigue syndrome	572 (20%)	427 (28%)	27 (9%)
PTSD	513 (20%)	445 (30%)	2 (<1%)
Suicide attempt	528 (20%)	429 (29%)	9 (3%)

Table 3 shows reported ACEs by exposure to sexual violence. More participants in the group exposed to sexual violence have grown up in families with alcohol problems, 30% compared with 15% among nonexposed; suicide attempts: 10% compared with 5% among nonexposed; or where they have witnessed violence: 6% and 10%, respectively, compared to 3% among the nonexposed.

**Table 3:** Adverse childhood experiences by exposure to sexual violence

	All types of sexual violence, *n* = 2606 (100%)	Exposed to penetrating sexual violence, *n* = 1500 (100%)	Not exposed to sexual violence, *n* = 291 (*n* = 100%)
Alcohol and drug problems in the family during upbringing	779 (30%)	483 (32%)	44 (15%)
Suicide attempts in the family during upbringing	266 (10%)	166 (11%)	15 (5%)
Witness to domestic violence during upbringing	147 (6%)	150 (10%)	9 (3%)

## 4 DISCUSSION

The proportion of depression, anxiety, and fatigue syndrome was about three times as common among participants exposed to sexual violence compared to nonexposed participants. Furthermore, PTSD was 100 times more common, and the suicide rate was 10 times as high among exposed compared to nonexposed participants. A doubled incidence of ACEs of alcohol problems, suicide attempts by family members or witnessed violence was also seen among the participants exposed to sexual violence compared with the nonexposed.

Women reported more sexual violence than men in the present study, 91% versus 40%. The fact that the women were more exposed than men is in line with previous data from epidemiological studies and reports, although the proportion exposed in both men and women in this study is well above numbers in earlier prevalence studies of sexual violence in the general population.^12^, ^13^ The higher proportion of exposed participants in this study is probably a result of the senders of the digital forms were individuals on e‐mail‐lists owned by organizations working with survivors of sexual violence.

The results, with more than a threefold proportion exposed to penetrative sexual violence who had or had been diagnosed with depression, anxiety or fatigue syndrome compared with not exposed, are in line with previous Swedish and international studies.^7^, ^8^, ^9^, ^14^ The fact that the proportion with suicide attempts is so high among participants exposed to sexual violence compared to unexposed is in line with both international studies and experience from WONSA's specialist clinic, where 38% of patients had made at least one suicide attempt, and 25% had made two or more suicide attempts.^10^, ^15^ This is also in line with results from a study on young people in the Stockholm Region, where a 26‐fold increased risk of seeking medical care due to suicide attempts was seen after a sexual assault had been registered in the medical record, compared with girls of the same age in the same residential area without such registration in the journal.^9^ The similarity regarding suicidality between the findings from consecutive patients at the WONSA clinic, the register‐based study and in the present online survey study suggest, that the survey had fairly good validity.

## 5 LIMITATIONS

The present study cannot deem causality on its own due to its cross‐sectional design, yet longitudinal studies have been conducted previously,^1^, ^16^, ^17^ indicating that causality exist, and the present study verifies the associations between ACEs and sexual violence with poor mental health and suicidality. The willingness to answer sensitive questions is a strength and indicates that the questions are formulated with knowledge of sexually violated people. In this report, however, 76% of the participants have post high school education, and 60% some degree of university education. According to Statistics Sweden (SCB), the corresponding figures for the Swedish population are 44% and 29%.^18^ This bias is important from a democratic perspective, but also since the prevalence of both exposure to violence and disease development can diverge between different socioeconomic groups. It is unclear whether the nonrepresentative level of education depends on the design or distribution of the survey. However, the fact that the questionnaires were disseminated through organizations that work with issues related to sexual violence has contributed to the uneven representativity of the sample. Another weakness is the uneven distribution between those exposed to or not exposed to sexual violence, which may have influenced the comparisons between the groups. However, the reporting of the incidence of diagnoses, suicide attempts and ACEs as percentages in each group means that valuable information emerges despite this. The fact that not everyone has answered all the questions makes it hard to analyze associations and the fact that sexual violence was not included among the questions concerning ACEs made it impossible to analyze differences in mean ACE‐scores between those exposed to sexual violence and unexposed. Another weakness is that there were no explicit questions about alcohol and drug addiction in the survey. As ACEs and sexual violence are risk factors for mental illness, suicidality and addiction problems, it would have been relevant to include questions on alcohol and drug use and dependency to further explore the association.

## 6 CONCLUSION AND FUTURE DIRECTIONS

The present study shows that it is possible to collect sensitive data on sexual violence and mental illness through digital surveys. This is important information for public health agencies in their mapping of the health of the population and will enable questionnaires that may better capture the health of the general population. One should focus on the results that are in line with already existing knowledge in parallel with continued knowledge acquisition and evaluation of how efforts for equal health affect exposure to sexual violence and ANDT problems. This applies, for example, to the fact that so many respondents report exposure already in childhood, and the increased proportion of sexual violence in individuals who grew up in families with alcohol problems, suicide attempts, or violence between guardians. In the same way, the health service's structural work to identify and be able to offer survivors of sexual violence effective treatment is an important part of the work for equal health.

Health care today receive criticism to the fact that it treats symptoms and not the root of the problem. We believe that ACE and sexual violence explain a large part of mental health problems of exposed individuals and that efforts should be made to identify and offer support to children in the risk zone for ACE and sexual violence as well as to children and adults who are or have been exposed to these pathogens. We need to make therapy for childhood trauma and sexual violence available to the broad public. Finally, steps should be taken to adapt the national suicide prevention strategy so that it will consider the association between sexual violence and suicide attempts, which has been so clearly demonstrated earlier in both international and national studies, and which is again is shown in the present study.

## AUTHOR CONTRIBUTIONS

**Axel C. Carlsson**: Methodology; writing – original draft; writing – review and editing. **Ulrika Owen**: Investigation; project administration. **Gita Rajan**: Conceptualization; data curation; formal analysis.

## ETHICS STATEMENT

Ethics permit was obtained by the local ethical committee in Stockholm: number 2019‐00481. Everyone who chose to participate received written information about the purpose of the survey and they gave consent before they had access to the questions. Written informed consent was obtained from the patient to publish this report in accordance with the journal's patient consent policy.

## TRANSPARENCY STATEMENT

The lead author Axel C. Carlsson affirms that this manuscript is an honest, accurate, and transparent account of the study being reported; that no important aspects of the study have been omitted; and that any discrepancies from the study as planned (and, if relevant, registered) have been explained.

## Data Availability

The data that support the findings of this study are available from the corresponding author upon reasonable request. The data that support the findings of this study are available from Facebook. Restrictions apply to the availability of these data, which were used under license for this study. Data are available from https://dataforgood.facebook.com with the permission of Facebook.
